# Trapping of ivermectin by a pentameric ligand-gated ion channel upon open-to-closed isomerization

**DOI:** 10.1038/srep42481

**Published:** 2017-02-20

**Authors:** Nurit Degani-Katzav, Moshe Klein, Moran Har-Even, Revital Gortler, Ruthi Tobi, Yoav Paas

**Affiliations:** 1 Laboratory of Ion Channels, The Mina and Everard Goodman Faculty of Life Sciences and The Institute of Nanotechnology and Advanced Materials, Bar-Ilan University, Ramat Gan 52900, Israel

## Abstract

Ivermectin (IVM) is a broad-spectrum anthelmintic drug used to treat human parasitic diseases like river blindness and lymphatic filariasis. By activating invertebrate pentameric glutamate-gated chloride channels (GluCl receptors; GluClRs), IVM induces sustained chloride influx and long-lasting membrane hyperpolarization that inhibit neural excitation in nematodes. Although IVM activates the *C. elegans* heteromeric GluClα/β receptor, it cannot activate a homomeric receptor composed of the *C. elegans* GluClβ subunits. To understand this incapability, we generated a homopentameric α7-GluClβ chimeric receptor that consists of an extracellular ligand-binding domain of an α7 nicotinic acetylcholine receptor known to be potentiated by IVM, and a chloride-selective channel domain assembled from GluClβ subunits. Application of IVM prior to acetylcholine inhibited the responses of the chimeric α7-GluClβR. Adding IVM to activated α7-GluClβRs, considerably accelerated the decline of ACh-elicited currents and stabilized the receptors in a non-conducting state. Determination of IVM association and dissociation rate constants and recovery experiments suggest that, following initial IVM binding to open α7-GluClβRs, the drug induces a conformational change and locks the ion channel in a closed state for a long duration. We further found that IVM also inhibits the activation by glutamate of a homomeric receptor assembled from the *C. elegans* full-length GluClβ subunits.

Ivermectin (IVM) is a macrocyclic lactone widely used as an anthelmintic drug to treat filarial diseases like onchocerciasis (river blindness) and elephantiasis (lymphatic filariasis) that afflict hundreds of millions of people worldwide^1^,^2^. IVM is also broadly used in cattle, swine and pets to kill gastrointestinal roundworms, lungworms, grubs, sucking lice and mange mites^3^. The high efficiency of IVM stems from its capability to activate invertebrate glutamate (Glu)-gated chloride channels (GluCl receptors; GluClRs) at nanomolar concentrations, and to keep the receptor’s ion channel continuously open^4^,^5^,^6^,^7^,^8^,^9^. Since the GluClRs are chloride selective, IVM causes sustained hyperpolarization across postsynaptic membranes, which inhibits vital functions in the worm like, locomotion^10^, pharyngeal muscle activity^11^,^12^,^13^ and secretion processes crucial for evading the host immune system^14^; reviewed by Geary and Moreno^15^ and Wolstenholme^16^.

GluClRs are pentamers belonging to the Cys-loop receptor superfamily^17^. As such, they share high primary, secondary, tertiary and quaternary structural homologies with other cationic or anionic Cys-loop receptors whose activating neurotransmitters are acetylcholine (ACh)^18^,^19^,^20^,^21^,^22^,^23^,^24^,^25^,^26^,^27^, serotonin^28^,^29^, γ-aminobutyric acid (GABA)^30^,^31^,^32^,^33^, glycine (Gly)^34^,^35^,^36^,^37^,^38^,^39^,^40^ or histamine^41^,^42^,^43^. Noteworthy, IVM can activate and/or potentiate a few vertebrate Cys-loop receptors, like GABA- and Gly-activated Cl^−^ channels^43^,^44^,^45^,^46^,^47^,^48^,^49^,^50^,^51^,^52^,^53^, and the α7-nicotinic AChR^54^,^55^, ae well as the P2X ATP-gated ion channels^56^,^57^,^58^,^59^, though with much higher concentrations than in GluClRs.

Genes (*glc-1* and *glc-2*) encoding two GluClR homologous subunits, GluClα (GLC-1; also named GluClα1) and GluClβ (GLC-2), were firstly cloned from the non-parasitic nematode *C. elegans*^4^. Later, additional genes encoding subunits of Glu-gated chloride channels were cloned from *C. elegans*^60^ and other invertebrates^61^ like parasitic worms^62^,^63^,^64^,^65^, insects^66^,^67^,^68^,^69^,^70^,^71^, crustaceans^72^, and mollusk^73^. In several cases, a single subunit was found to form a functional homomeric receptor–channel that can be gated by both Glu and IVM independently. For example, the GluClα2 (AVR-15) subunit of *C. elegans*^6^, the DrosGluCl-α subunit of *Drosophila melanogaster*^74^, the GluClα2B subunit of *H. contortus*^75^, the MdGluClα subunit of *Musca domestica*^66^, the GluCl exon-3 variants of *Bombyx mori*^68^, and the AgGluCl-a1 of *Anopheles gambiae*^70^. In contrast, when expressed in *Xenopus* oocytes, the *C. elegans* GluClα subunit (GLC-1) forms homomeric receptors that can be activated by IVM but not by Glu, whereas the *C. elegans* GluClβ subunit (GLC-2) forms homomeric receptors that can be activated by Glu but not by IVM^4^,^7^,^8^,^76^. On the other hand, a heteromeric GluClR consisting of the *C. elegans* α (GLC-1) and β (GLC-2) subunits can be activated by both Glu and IVM independently^4^,^5^,^6^,^7^,^8^,^9^.

A three-dimensional (3-D) crystal structure of a truncated homomeric GluClα receptor (GluClα_cryst_R, PDB 3RIF) indicates that IVM binds at the α/α intersubunit interfaces in the ion-channel pore periphery^77^. A recent study indicates that incorporation of the GluClβ subunit in *C. elegans* GluClR assemblies confers increased receptor sensitivity to IVM^78^. Taken together with a recent determination of the subunit stoichiometry and arrangement in a *C. elegans* heteromeric GluClα/βR^79^, it seems reasonable that IVM binds at GluCl β/α intersubunit interfaces. So, if IVM can interact with the GluClβ subunit to activate the *C. elegans* heteromeric GluClα/βR, then why can it not activate the homomeric GluClβR ? To answer this question, we have first analyzed the effects of IVM on a highly expressed chimeric α7-GluClβR whose extracellular ligand-binding domain binds ACh to gate a transmembrane ion-channel pore adopted from the *C. elegans* GluClβR. Subsequently, we determined the effect of IVM on homomeric receptors assembled from the full-length *C. elegans* GluClβ subunit.

## Results

### Structural and basic functional properties of the chimeric α7-GluClβ receptor

**Figure 1:**
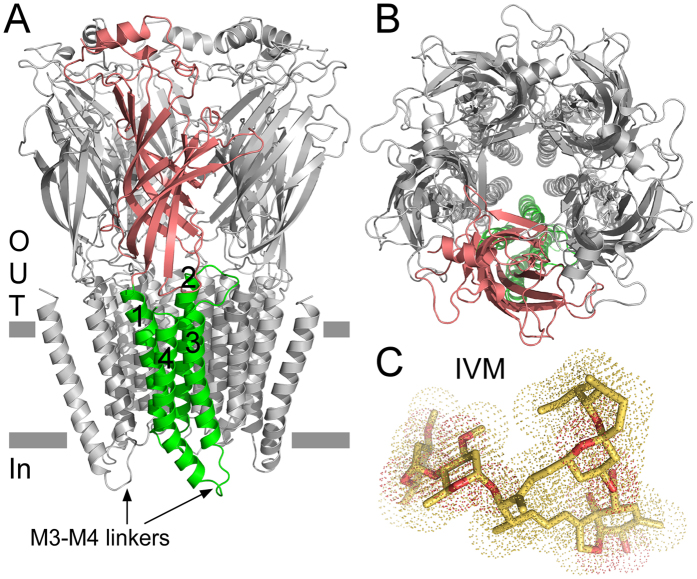
Structural properties of the chimeric α7-GluClβR. (**A**) Side view of a three-dimensional (3-D) homology model of the chimeric α7-GluClβR. For clarity, one of the five subunits is colored as follows. The N-terminal segment (reddish) forms the extracellular ligand-binding domain upon receptor assembly. The C-terminal segment (green) has four transmembrane helices (numbered 1 to 4) and it forms the ion-channel pore domain upon receptor assembly. The M3–M4 linkers of two adjacent subunits are indicated; they are much longer in the experimented receptor, but their structure is missing, as it is unavailable in the atomic-scale template used for homology modelling. (**B**) Top view of the α7-GluClβR homology model showing five identical subunits, organized in a five-fold symmetry around the axis of ion conduction that is perpendicular to the viewer. (**C**) Three dimensional structure of ivermectin. Carbon and oxygen atoms are colored in yellow and red, respectively.

In this study, we have used a chimeric Cys-loop receptor that consists of five identical subunits. Each subunit is a chimera generated by fusing the N-terminal extracellular sequence of the neuronal α7 nicotinic acetylcholine receptor (α7-nAChR) subunit to the C-terminal transmembrane and intracellular sequence of the *C. elegans* GluClβ subunit (GLC-2) (Supplementary Fig. S1)^80^,^81^. This chimeric α7-GluClβ Cys-loop receptor robustly responds to ACh that binds to the extracellularly facing ligand-binding domain (LigBD) to open a Cl^−^-selective ion channel pore^80^,^81^. Figure 1A and B, presents a homology model of the chimeric α7-GluClβR. Figure 1C depicts a 3-D structure of IVM.

**Figure 2:**
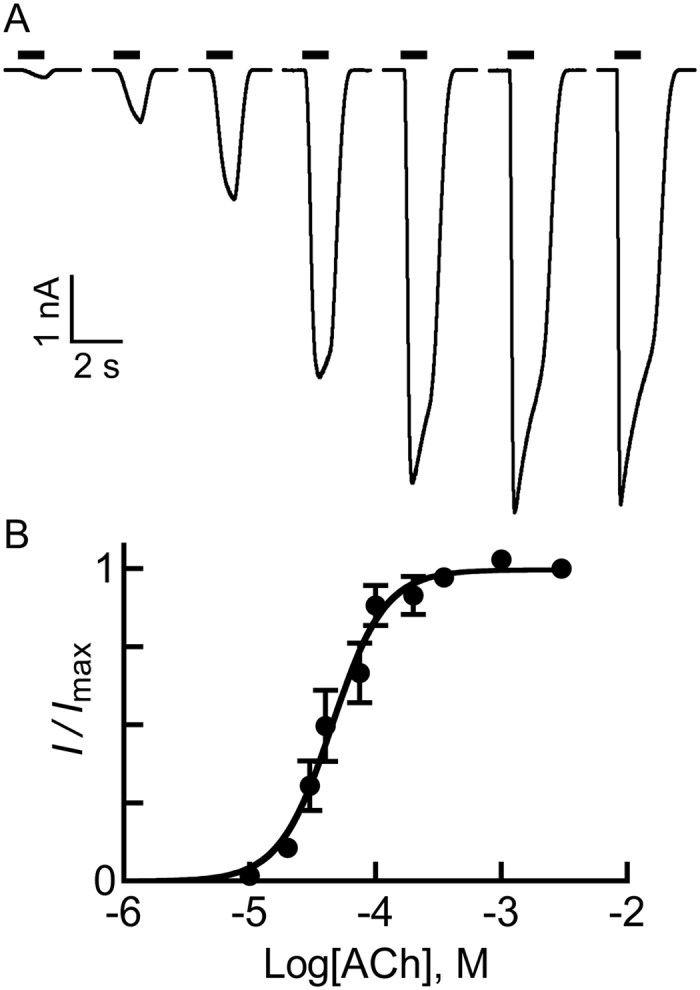
ACh-activation properties of the chimeric α7-GluClβR. (**A**) Representative current traces evoked by ACh in Chinese hamster ovary (CHO) cells expressing the chimeric α7-GluClβR. ACh was applied for 1 sec at micromolar concentrations of: 10, 20, 30, 75, 200, 1000 and 3000 (black bars). Recordings were performed at −60 mV. (**B**) A dose-response curve plotted for the responses of the α7-GluClβR to ACh. Data was extracted from experiments performed as in panel A. The curve was fitted to the averaged data points with a non-linear regression using the Hill equation 1 (Eq. 1, see “Materials and Methods”) (*r*^2^ = 0.996). Error bars correspond to SEM (N = 12).

Figure 2 shows representative ACh-evoked currents and a dose-response curve indicating an ACh-EC_50_ value of 46 ± 14 μM and a Hill coefficient of 2.55 ± 0.3. These values are very close to previous determinations^81^.

### Application of IVM prior to ACh inhibits the responses of the α7-GluClβR

IVM can activate the *C. elegans* homomeric GluClα and heteromeric GluClα/β receptors expressed either in *Xenopus* oocytes^4^,^5^,^6^,^7^,^8^,^61^,^82^, HEK cells^78^ or CHO cells^79^, but it cannot activate the *C. elegans* homomeric GluClβR expressed in these cells^4^,^7^,^8^,^76^,^78^,^79^. Since, in the GluClα_cryst_R, IVM binds at the periphery of the ion-channel pore domain^77^, we reasoned that IVM would not activate the chimeric α7-GluClβR whose ion-channel pore domain is that of the GluClβR. On the other hand, previous studies performed with the α7-nAChR showed that IVM potentiated the ACh-elicited currents and decreased the ACh-EC_50_ by 20 fold^54^,^55^. Hence, we were wondering whether IVM would be able to potentiate the response of the chimeric α7-GluClβR to ACh. Our curiosity emerged since, in the GluClα_cryst_R, IVM forms a few van der Waals interactions with the M2-M3 loop^77^ that is located at the interface between the LigBD and the pore domain and interacts with entities of the LigBD to gate Cys-loop receptors. For example, in the GABA_A_R^83^,^84^,^85^, GlyR^86^,^87^, 5HT3_A_R^88^,^89^,^90^, muscle nAChR^91^,^92^,^93^, neuronal α7-nAChR^94^,^95^,^96^, as well as in the chimeric α7-GlyR^97^and α7–5HT3_A_R^98^.

**Figure 3:**
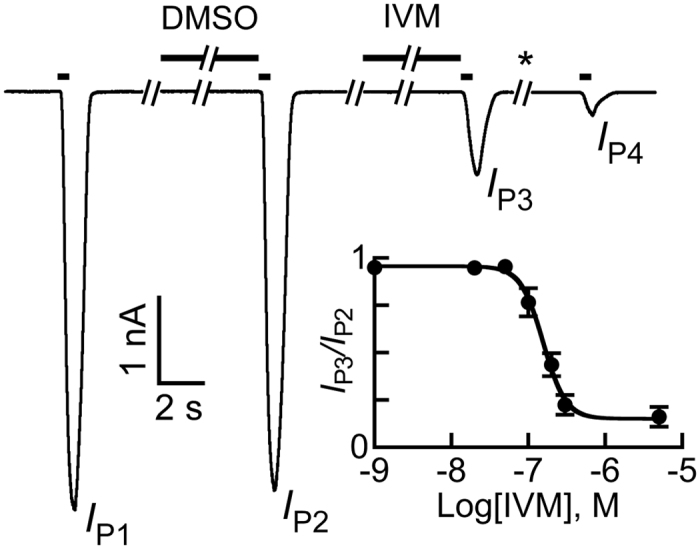
Inhibitory effect of IVM pre-application on the response of the α7-GluClβR to ACh. Following the first ACh application, which gave peak *I*_P1_, the cell was washed with a physiological solution for 40 sec. Then, as a control, 0.01% DMSO was applied for 10 sec prior to the second ACh application that gave peak *I*_P2_. Next, following a 40-sec-long wash, 5 μM IVM (in the presence of 0.01% DMSO) was applied for 10 sec before its replacement by ACh (giving peak *I*_P3_). Finally, following a 2-min-long wash (*), ACh was applied again (giving peak *I*_P4_). In all applications, 50 μM ACh was applied for 0.5 seconds. Inset, a curve corresponding to the inhibition of ACh (50 μM)-elicited currents by increasing concentrations of IVM. Data were extracted from experiments performed as in panel A. Since the currents could not be recovered following an exposure to IVM, each cell was exposed only to one concentration of IVM. Then, to provide the normalized ACh response, the peak current recorded in response to ACh immediately after IVM application (i.e., *I*_P3_) was divided by the peak current recorded in response to ACh immediately after DMSO application (i.e., *I*_P2_). The curve was fitted to the averaged data points with a non-linear regression using the Hill equation 2 (Eq. 2; *r*^2^ = 0.996). Error bars correspond to SEM. Total of 41 cells were used to obtain the averaged points. Recordings were performed at −60 mV.

Figure 3 shows robust responses to ACh alone (*I*_P1_) or to ACh applied immediately after pre-application of 0.01% DMSO (the content in the IVM-containing solution) (*I*_P2_). The DMSO-containing solution had no significant effect on the current amplitude (*I*_P2_*/I*_P1_ of 95 ± 1.8%; *P* > 0.05, N = 42, paired, two-tailed Student’s *t*-tests). In contrast, pre-application of 5 μM IVM for 10 sec reduced the ACh-elicited response to 16 ± 5.2% of the response obtained immediately after the DMSO pre-application (percent *I*_P3_*/I*_P2_; *P* < 0.001, N = 8) (Fig. 3). Notably, the initial responses to ACh could not be recovered even after a long-term wash (* > 2 min) (e.g., Fig. 3, *I*_P4_), and their magnitude was 4.4 ± 1.3% of the responses obtained after the DMSO pre-application (percent *I*_P4_*/I*_P2_; *P* < 0.001, N = 5).

Since the ACh-evoked response could not be recovered after exposure of the cell to IVM, we had to perform the above application protocol in different cells for each IVM concentration separately. By that, we obtained the inhibition graph shown in the inset of Fig. 3. The IVM-IC_50_ was found to be 156 nM, and the Hill coefficient ∼3.

**Figure 4:**
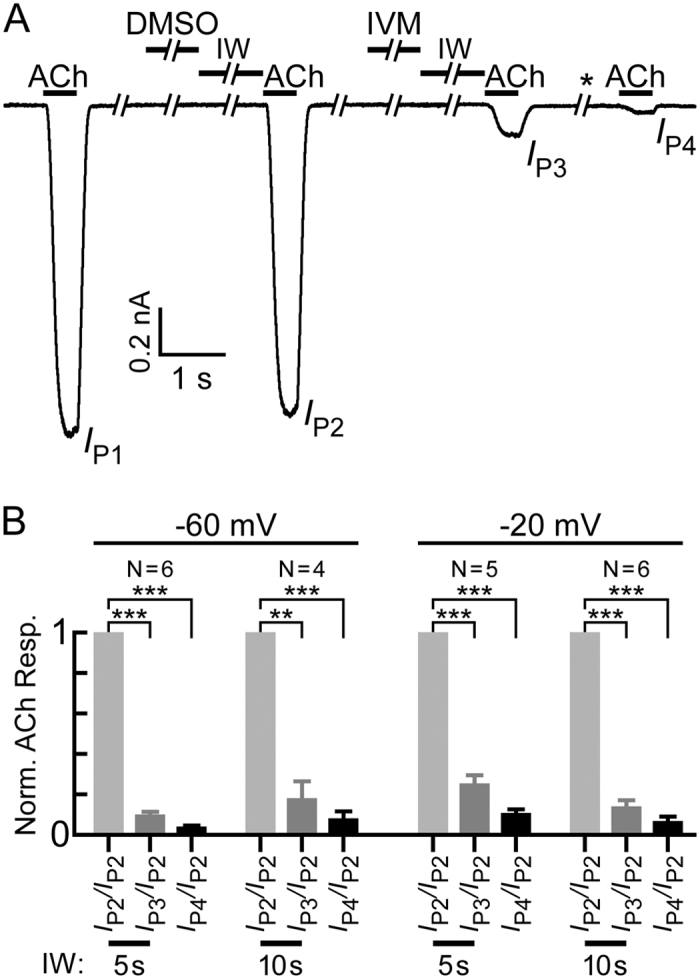
Washes introduced between the IVM and ACh applications do not prevent the inhibition by IVM. (**A**) A representative current trace evoked by ACh to give: a first peak (*I*_P1_); a second peak (*I*_P2_) after the DMSO application and an intermediate wash (with a physiological solution, IW); a third peak (*I*_P3_) after the IVM application and an intermediate wash (IW); and a fourth peak (*I*_P4_) after further long-term wash (* > 100 sec). In all cases, 50 μM ACh was applied for 0.5 seconds. DMSO (0.01%) and IVM (5 μM; containing 0.01% DMSO) were applied for 4 seconds. Washes performed immediately after the first and second ACh applications were 40-sec long (not indicated by a bar above the trace). Intermediate washes (IW) were applied for 5 seconds. Recording of this specific trace was performed at −60 mV. (**B**) Histograms of data extracted from experiments performed as in A, which include a 4-sec-long pre-application of IVM (5 μM), followed by a 5- or 10-sec-long intermediate wash (IW) and an ACh (50 μM) application (for 0.5 sec), at −60 or −20 mV membrane voltages. Each current peak (*I*_P3_ or *I*_P4_) was normalized to the current peak recorded after the application of 0.01% DMSO (*I*_P2_) in the same cell (Norm. ACh Resp. stands for normalized acetylcholine response). Each cell was exposed to IVM only once, because the currents could not be recovered even after a long-term wash. The bars correspond to the averaged normalized currents with error bars corresponding to SEM of N cells, as indicated within the figure. The time of the intermediate wash (IW) is indicated at the bottom. Paired, two-tailed Student’s *t*-tests indicate that ***P < 0.0005 and ** 0.0005 < *P* < 0.005. Two-way analysis of variance (ANOVA) between the *I*_P3_*/I*_P2_ ratios obtained following 5-sec-long or 10-sec-long intermediate washes, at −60 mV or −20 mV, indicates that there is no difference between the extent of inhibition observed under these four conditions (*P* > 0.05). The same two-way ANOVA probability (*P* > 0.05) was obtained for the *I*_P4_*/I*_P2_ ratios.

The results in Fig. 3 may indicate that IVM binds to the resting (closed) state and cannot dissociate from the receptor during the application of ACh. We however cannot exclude the possibility that the immediate application of ACh after the application of IVM stabilized the receptor in a pre-open state for a long duration. We therefore changed the application protocol and introduced intermediate washes between the IVM and ACh applications. Clearly, neither 5- nor 10-sec-long intermediate washes could prevent the strong inhibitory effect of IVM pre-application (*I*_P3_ and *I*_P3_*/I*_P2_ in Fig. 4A and B, respectively). Furthermore, a long-term wash introduced after the first recovery trial (Fig. 4A, asterisk), was followed by even a greater inhibitory effect (*I*_P4_ and *I*_P4_*/I*_P2_ in Fig. 4A and B, respectively).

Since the GluClα_cryst_R indicates that IVM is accommodated at the protein-lipid interface^77^, we examined the effect of the membrane voltage on the IVM inhibitory effect. Similar effects have been observed at −60 mV and −20 mV (Fig. 4B), suggesting that the electric field of the membrane does not influence IVM association with the chimeric α7-GluClβR.

### IVM accelerates the decline of ACh-elicited macroscopic currents in cells expressing α7-GluClβ receptors

**Figure 5:**
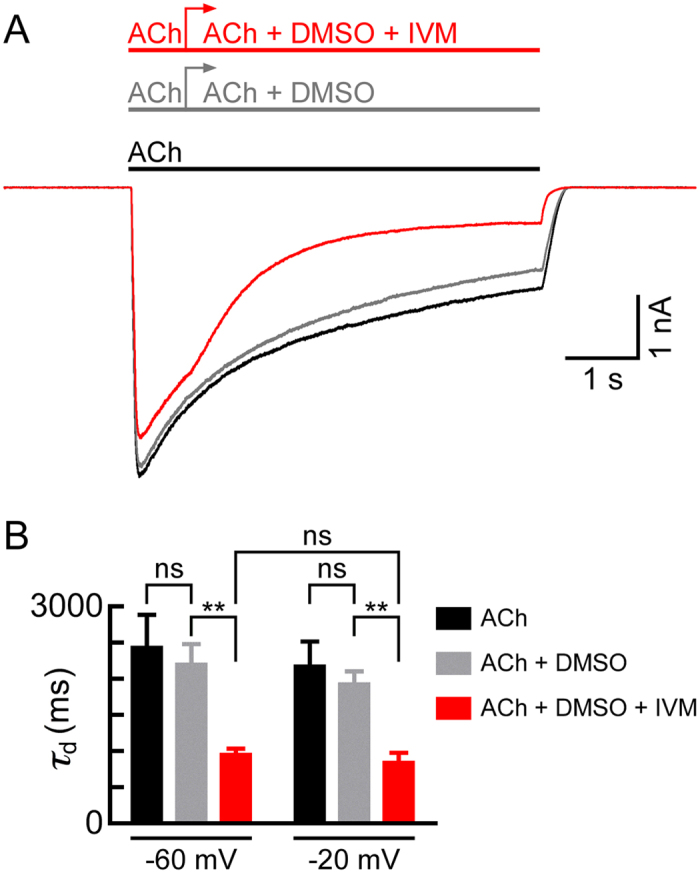
IVM accelerates the current decline when added after receptor activation by ACh. (**A**) Representative currents elicited first by ACh alone (350 μM) that was applied for 5.5 sec (black bar and trace). Following a wash of a minute, the same cell was challenged again with ACh (350 μM), and after reaching to the peak (i.e., after 0.75 s), 0.01% DMSO was added, as a control (gray bar, arrow and trace). Following a further wash (for a minute), the same cell was challenged again with a 5-μM-IVM solution (in 0.01% DMSO; red bar, arrow and trace). Recordings were performed at −60 mV. (**B**) Time constant of current decline (*τ*_d_). Values of *τ*_d_ were determined by exponential fits to the declining phase of the currents using Eq. 3. Data was extracted from experiments performed as in (**A**) (ACh, 350 μM; DMSO, 0.01%; IVM, 5 μM). In all three cases, the kinetics was measured at the same time range; starting ~0.75 sec after ACh application (the time of DMSO or IVM addition) and ending at the time of wash application. Error bars correspond to SEM of N = 6 and N = 5 cells for recordings at −60 mV and −20 mV, respectively. Paired, two-tailed Student’s *t*-tests indicate that for not significant (“ns”) *P* > 0.3, and ** 0.001 < *P* < 0.005.

Application of 350 μM ACh (saturating concentration) for 5.5 sec on cells expressing the chimeric α7-CluClβR, resulted in currents that rapidly reached a peak and then declined to 31 ± 8.6% (N = 5) of the peak amplitude (Fig. 5A, black trace). We took advantage of the slow and partial current decline observed here, and examined the capability of IVM to associate with the α7-GluClβRs after activation, during the phase of current decline. To this end, immediately after reaching to the ACh-evoked peak, we added 5 μM IVM (dissolved in 0.01% DMSO) to the ACh-containing solution. The red colored current trace in Fig. 5A clearly indicates that IVM accelerates the current decline, and reduces the amplitude of the steady-state current. The grey current trace (Fig. 5A) corresponds to a control showing that 0.01% DMSO does not substantially change the response to ACh alone. Statistical analysis of the time constant of current decline (*τ*_d_) indicates that DMSO alone had no significant effect on *τ*_d_, neither at −60 mV nor at −20 mV (Fig. 5B). In contrast, IVM reduced the *τ*_d_ values by more than two folds at both membrane voltages, −60 mV and −20 mV, to a statistically similar extent (Fig. 5B). These results reinforce our above suggestion that the electric field of the membrane does not influence IVM association with the chimeric α7-GluClβR.

### How does IVM decrease Cl^−^ ion flow through the chimeric α7-GluClβR?

**Figure 6:**
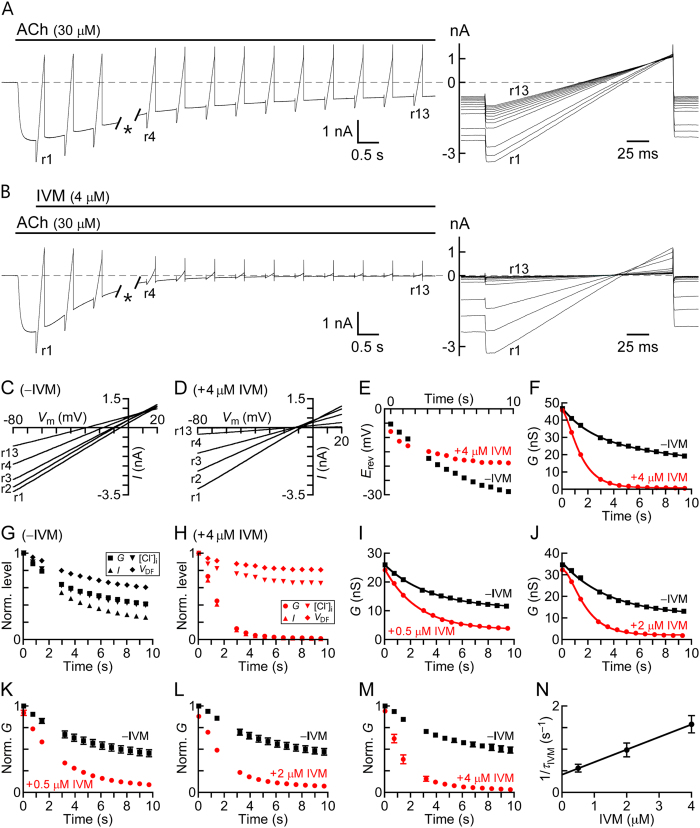
Chloride conductance of α7-GluClβRs. Representative whole-cell current traces elicited by: (**A**) ACh alone and (**B**) ACh to which IVM was added after the current peak. Recordings were performed at −60 mV with intervening 200-ms-long voltage ramps applied from −80 to +20 mV during the current decline, giving output currents r1–r13. *, time lapse of ~1 sec between protocols’ exchange (see “Supplementary Materials and Methods”). Traces in A and B were recorded from the same cell, with an intermediate 74-sec-long wash. Right panels show superimposition of the output currents. (**C,D**) Current-voltage (*I/V*) relations extracted from A and B, respectively (r13 in D overlaps the x-axis). (**E**) Reversal potentials (*E*rev) extracted from the voltage-ramps’ output currents of A (black squares) and B (red circles), plotted as a function of time (0 sec, time of r1). (**F**) Decay of chloride chord conductance plotted as a function of time, in the absence or presence of IVM. The conductance points were calculated at −65 mV based on the voltage-ramps’ output currents shown in A and B. Exponential fits (Eq. 6) are shown. (**G,H**) Normalized (Norm.) conductance (*G*), current (*I*), intracellular Cl^−^ ion concentration ([Cl]_i_), and electrochemical driving force (*V*_DF_) plotted as a function of time for ACh-dependent responses in the absence (panel G) or presence (panel H) of IVM (relating to responses in A and B, respectively). (**I,J**) Decay of chloride chord conductance plotted as a function of time in representative cells exposed to different IVM concentrations. (**K,L,M)** Averaged normalized chloride chord conductance (Norm. *G*) plotted as a function of time under different IVM concentrations. In each cell, each conductance was normalized to the conductance extracted from r1 of the ACh-alone application. N = 7, 11 and 8 in K, L and M, respectively. Error bars are SEM. (**N**) 1/*τ*_IVM_ plotted as a function of IVM concentration, using equation 7 for curve fitting by linear regression (*r*^2^ = 0.999). 26 cells were analyzed to obtain the averaged 1/τ_IVM_ points. Error bars are SEM. In all panels, ACh concentration is 30 μM.

The α7-nAChR is a fast desensitizing receptor whose current response rapidly declines in the presence of saturating ACh concentrations due to channel closing^96^,^98^,^99^,^100^,^101^,^102^,^103^,^104^,^105^,^106^,^107^,^108^,^109^,^110^. Compared with the α7-nAChR, in the case of the chimeric α7-GluClβR studied here, the current declines much slower under saturating ACh concentration (e.g., Fig. 5A, black trace). Therefore, over a relatively long-term recording of inward currents through the Cl^−^-selective α7-GluClβR, the driving force acting on Cl^−^ ions may decrease due to the outflow of Cl^−^ ions and the decrease in their intracellular local concentration near the channels during the recording time window. A decrease in intracellular Cl^−^ ion concentration in the vicinity of the channels has already been suggested for slowly-desensitizing GABA and Gly Cys-loop receptors^111^. To distinguish between desensitization (channel closing) and a decrease in the driving force acting on Cl^−^ ions, we activated the receptors with 30 μM ACh, and after the current reached to its peak, we performed voltage ramps throughout the current-declining phase, as has been performed previously^111^. Figure 6A presents an example for such an experiment, where the output currents of the voltage ramps are obtained in the absence of IVM. Superimposition of the voltage-ramps’ output currents shows a moderate decrease in slopes (Fig. 6A, right). The current-voltage relations (*I/V* curves) extracted from the output currents (Fig. 6C) indicate that, during the application of ACh alone (in the absence of IVM), the reversal potential (*E*_rev_) decreases from −5.3 mV to −28.9 mV. The black squares in Fig. 6E correspond to the *E*_rev_ values determined for each voltage ramp when ACh is applied alone. The leftward (negative) shift in the *E*_rev_ values observed in the absence of IVM (∆*E*_rev_ = 23.6 mV; Fig. 6C and E, black squares) reflects a change in the Nernst potential for Cl^−^ ions. By using the experimental *E*_rev_ values and the Nernst equation (Eq. 4), we calculated the intracellular concentrations of Cl^−^ ions ([Cl]_i_) and found that it is 122.7 mM at the beginning and 48.9 mM at the end of the current-decline phase (r1 and r13 in Fig. 6A, respectively). Therefore, when measuring inward Cl^−^ currents over 9.6-sec-long applications of ACh alone (e.g., Fig. 6A), a significant component of the current decline can be attributed to a decrease in the electrochemical driving force (*V*_DF_) acting on Cl^−^ ions (*V*_DF_ = *V*_m_ − *E*_rev_). *V*_DF_ probably decreases because Cl^−^ ions flow out of the cell, and [Cl]_i_ in the vicinity of the channels decreases with no capability to recover within the time window of the recording.

We applied the same experimental protocol on the representative cell shown in Fig. 6A, but in the presence of IVM that was added immediately after the current reached to its peak (Fig. 6B). In this experiment, we used 4-μM-IVM solution that contained 0.008% DMSO. Superimposition of the voltage ramps’ output currents recorded in the presence of IVM shows a sharper decrease in slopes (Fig. 6B, right) than in the absence of IVM (Fig. 6A, right). The *I/V* relations indicate that, within the recording time in the presence of IVM, the reversal potential (*E*_rev_) decreases from −7.9 mV to −18.9 mV (Fig. 6D). The leftward (negative) shift in the reversal potential observed in the presence of IVM (∆*E*_rev_ = 11 mV; Fig. 6D and E, red circles) reflects a much smaller change in the Nernst potential for Cl^−^ ions, as compared with the shift observed in the absence of IVM. The *I/V* relations allowed us to determine the change in the chloride conductance throughout the 9.6-sec application time. For that, we have calculated the chloride chord conductance at −65 mV (Eq. 5), which turned to be virtually the same as the chloride chord conductance at +15 mV. Figure 6F shows exponential curves fitted to the chord conductance points (Eq. 6) that were calculated from the currents shown in Fig. 6A and B. Clearly, in the presence of 4 μM IVM, the chloride conductance declines much faster (red curve) than in the absence of IVM (black curve) (Fig. 6F). Using the experimental *E*_rev_ values, we calculated the [Cl]_i_ and the *V*_DF_ for each voltage ramp output current at −65 mV, as has been performed above for the ACh application in the absence of IVM. While the changes in [Cl]_i_ and *V*_DF_ were slower and weaker in the presence of IVM (Fig. 6H) than in the absence of IVM (Fig. 6G), the decline of the conductance and current was much faster and stronger in the presence of IVM (Fig. 6H) than in the absence of IVM (Fig. 6G). Moreover, the current decline and the conductance decline in the presence of IVM overlap. Hence, we suggest that the conductance decline observed in the presence of IVM reflects structural changes in the ion conductance pathway, which eventually prevent Cl^−^ ions to cross the ion-channel pore.

To determine the kinetics of conductance decline at lower IVM concentrations we had to use different cells since we could not reproduce the initial robust response (the one shown in Fig. 6A) after the exposure of the cell to IVM, even following a long-term wash (70 seconds) (Supplementary Fig. S2). Figure 6I and J shows the conductance decline in representative cells under IVM concentrations lower than 4 μM. The entire analysis described in this section was applied to 26 cells; and the averaged normalized conductance decline, under three different IVM concentrations, is presented in Fig. 6K–M.

We then took advantage of the capability of IVM to accelerate the current decline in order to determine the binding affinity of IVM. To this end, the voltage ramps’ output currents obtained in the presence of IVM were subtracted from their counterpart currents obtained in the absence of IVM. Then, we calculated the chloride conductance based on the resulting subtracted currents at each ramp. This chloride conductance is hereafter termed *G*_IVM_. In each experimented cell, we fitted exponential curves to the first 5-6 conductance points, where there was no substantial difference between the ∆*E*_rev_ values in the absence or presence of IVM. These exponential fits for the *G*_IVM_ points were obtained at various IVM concentrations, and they actually provide the time constant of the IVM-dependent Cl^−^-conductance decay (*τ*_IVM_). Figure 6N shows that the averaged reciprocal *τ*_IVM_ values increase linearly with the increase in IVM concentrations.

IVM does not readily dissociate from the receptor (Supplementary Fig. S2) and the number of possible intermediate IVM-bound states is not known. Hence, the simplest possible kinetic model that could describe the acceleration of current decline in the presence of IVM would be one in which an ACh-activated conducting receptor (R*_ACh_) closes a gate in the ion-channel pore upon IVM binding (IVM**·**R_ACh_), as follows:


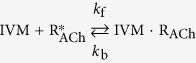


Figure 6N shows a curve fitted to the 1/*τ*_IVM_ points by a linear regression using equation 7. The slope of this curve corresponds to the IVM association rate constant (*k* forward, *k*_f_ = 290800 M^−1^⋅s^−1^). The IVM dissociation rate constant (*k* backward, *k*_b_ = 0.41_ _s^−1^) is the extrapolated intercept of the linear curve with the y-axis in Fig. 6N. The *K*_d_ (*k*_b_/*k*_f_) for IVM binding to an activated α7-GluClβ chimeric receptor would therefore be 1.4 × 10^−6^ M. This *K*_d_ value is nine times higher than the IC_50_ value observed when IVM is applied prior to activation (156 nM; Fig. 3).

### IVM becomes trapped during the receptor open-to-closed isomerization

**Figure 7:**
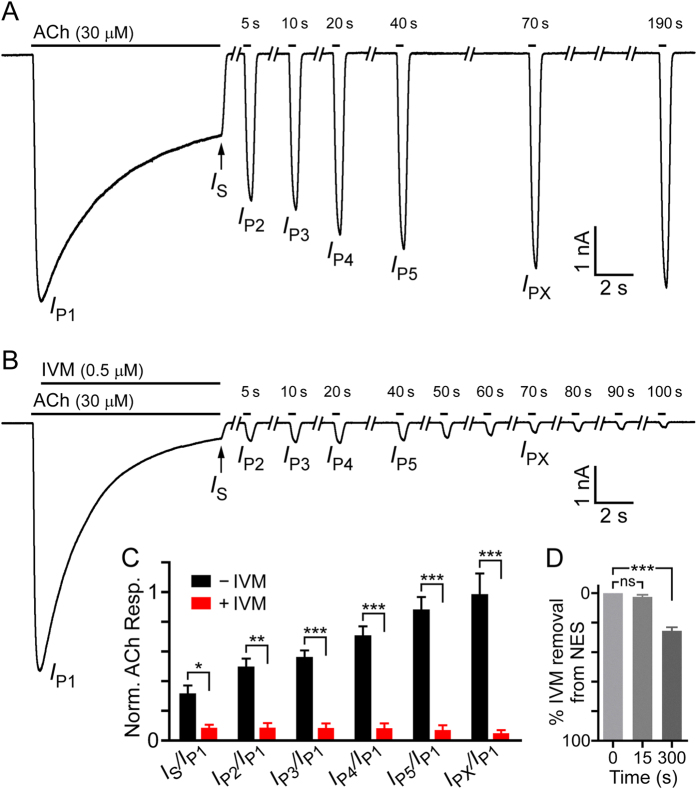
Recovery of ACh-evoked currents in the absence or presence of IVM. (**A**) Representative currents elicited by a long-term application (9.6 sec) of 30 μM ACh to obtain peak *I*_P1_, followed by repetitive short-term applications (350 ms) of 30 μM ACh (black bars above the current traces) with intermediate washes. Times indicated above the short bars were measured from the end of the initial, long-term ACh application (that gave *I*_P1_ and *I*_S_). (**B**) Representative currents for the same experiment as in A, but with IVM (0.5 μM) added after peak *I*_P1_. Currents in A and B were measured at −60 mV. (**C**) Normalized ACh response (y-axis) calculated by the current peak ratios that are indicated below the x-axis. Current peak ratios were extracted from experiments like those shown in A and B. Black bars correspond to results obtained with ACh alone, as in panel A (N = 8). Red bars correspond to ACh-elicited currents where IVM was added as in panel B (N = 5). Error bars correspond to SEM. Asterisks correspond to unpaired, two-tailed Student’s *t*-tests with 0.001 < *P < 0.05, 0.0001 < **P < 0.001 and ***P < 0.0001. (**D**) Fractional removal of IVM from the aqueous phase of a 1-ml solutions containing 140,000 CHO cells and 4 μM IVM. Removal of IVM was determined following the indicated incubation times by measuring the absorbance of supernatants at 245 nm wavelength (e.g., Supplementary Fig. S3; Supplementary Materials and Methods). Note that, no IVM removal is indicated at the top of the y-axis. Mean ± SEM of seven independent experiments are presented. Paired, two-tailed Student’s *t*-tests indicate that for “ns” (not significant) *P* > 0.2, and ***P < 0.0001.

The residency time for a drug bound to its target is provided by 1/*k*_b_^112^. The results of the previous section indicate that, for IVM, the residency time is ~2.5 sec, which means that IVM should have been removed within a wash of a few seconds. However, as said, the initial robust ACh-elicited current response (e.g., Fig. 6A) could not be reproduced following cell exposure to IVM, even after a wash lasting 70 seconds (Supplementary Fig. S2). To better understand the effect of IVM, we applied ACh (alone) for 9.6 seconds and then re-applied the same ACh concentration (alone) several times, each for 350 millisecond. Figure 7A and C (black bars) shows that when re-applications were performed 5, 10, 20 and 40 seconds after the end of the initial (long-term) ACh-application, the current amplitudes did not fully recover. The unrecovered current portion may indicate that the [Cl]_i_ near the channels and the *V*_DF_ at these time points have not been restored to initial values. We however do not exclude the possibility that part of the current is missing due to acute desensitization^113^, which may result in reversible stabilization of the receptor in a deeply desensitized (closed) state - as has been suggested for nAChRs in midbrain dopamine neurons^114^ and recombinant α4β2 neuronal nicotinic AChRs^115^ (reviewed by Giniatullin *et al*.^116^). Figure 7A and C (black bars) also shows that it takes at least 70 seconds for the macroscopic current amplitude to recover. This recovery excludes the possibility of functional rundown since there was no permanent inactivation as described, for example, in recombinant α3β2^117^, α4β2^118^ and α7^119^ nAChRs. When IVM was combined with ACh after the current reached to its peak in the long-term application, the current amplitude could not be recovered even after 100 sec of wash (Fig. 7B and C, red bars).

**Figure 8:**
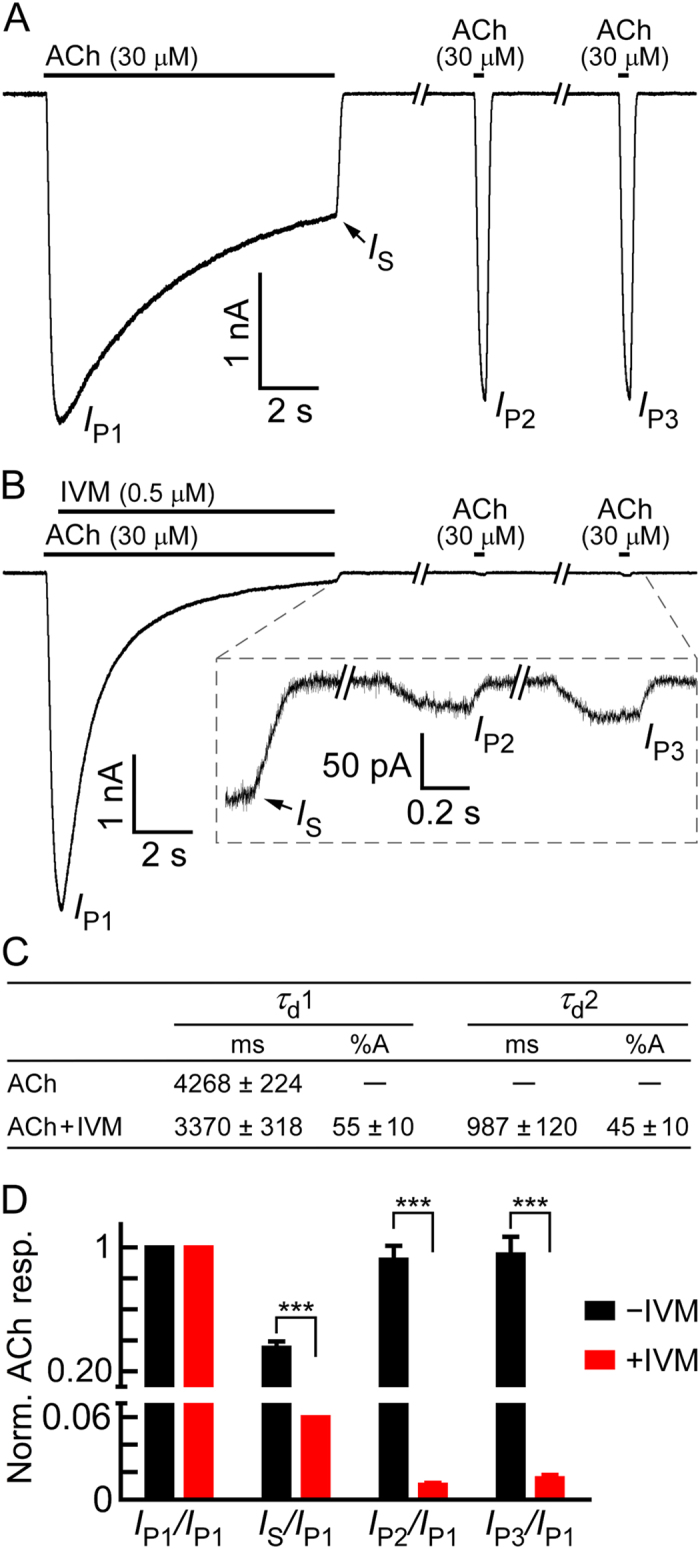
The inhibition of ACh-elicited currents by addition of IVM is irreversible. (**A**) Representative currents elicited by a long-term application (9.6 sec) of ACh followed by two short-term (350 ms) applications of ACh, with intermediate long-term washes, each lasting 3 minutes. (**B**) Representative currents for the same experiment as in A, but with the exception of IVM (0.5 μM) addition after the ACh-elicited current reached to its peak (*I*_P1_). Inset, magnification of the indicated segment of the current trace. In both panels A and B, black bars above the current traces indicate ACh or IVM applications. (**C**) Kinetics of current decline under long-term application (9.6 sec) of 30 μM ACh, in the absence or presence of 0.5 μM IVM (performed as described in A and B). In both cases, the application of ACh alone or the co-application of ACh with IVM, the kinetics of current decline was measured from 0.75 sec after the ACh application started until the end of the application. %A stands for the amplitude percentage. Error bars correspond to SEM of N = 10 cells per each condition (ACh alone or ACh + IVM). (**D**) Normalized ACh response (y-axis) calculated by the current peak ratios that are indicated below the x-axis. Current ratios were extracted from experiments performed as shown in A and B. Black bars correspond to experiments where all applications contained only ACh (e.g., panel A; N = 10). Red bars correspond to experiments where IVM was added during the initial, long-term application of ACh (e.g., panel B; N = 10). Error bars correspond to SEM. Unpaired, two-tailed Student’s *t*-tests indicate *** *P* < 0.0001.

To examine if IVM can be removed from the receptor by longer wash periods, we extended the washing time. Figure 8A shows a control experiment, where ACh was applied alone for 9.6 seconds followed by two additional short-term ACh applications (350 milliseconds), with two intermediate washes each lasting 3 minutes. As can be seen in Fig. 8A, the initial (long-term) ACh application resulted in a response that reached a peak (*I*_P1_) and declined with one exponential time course (*τ*_d_1, Fig. 8C). The current remaining at the end of the initial ACh application was 35 ± 4% of the initial peak (percent *I*_S_*/I*_P1_ in Fig. 8D, black bars). Figure 8A and D (black bars) also shows that the initial current amplitude (*I*_P1_) was completely recovered (*I*_P3_*/I*_P1_). In contrast, when IVM was added to ACh immediately after the current reached to its peak (Fig. 8B, leftmost response), the current declined with double exponential time course. One of the two time constants (*τ*_d_2) is significantly smaller than the time constant measured in the presence of ACh alone (Fig. 8C; *P* < 0.0001, unpaired, two-tailed Student’s *t*-test). In addition, the steady-state current (*I*_S_) was found to be 6% ± 0.01% of the initial amplitude, significantly smaller than in ACh alone (Fig. 8D, percent *I*_S_*/I*_P1_, red bar vs black bar). Furthermore, very small responses could be seen upon re-application of ACh alone after 3 and 6 minutes of wash (Fig. 8B, inset; Fig. 8D, *I*_P2_/*I*_P1_ and *I*_P3_/*I*_P1_ in red). Since, in the control experiments, the current could be recovered almost completely after a 3-min wash (Fig. 8A, *I*_P2_; Fig. 8D, *I*_P2_*/I*_P1_ in black), we conclude that the [Cl]_i_ in the vicinity of the channels and *V*_DF_ largely recovered within the 3-min wash. When IVM is added, the recovery of the [Cl]_i_ and *V*_DF_ is expected to take place even sooner, because compared with the control, the current declines much faster to *I*_S_ and *I*_S_*/I*_P1_ is much lower (Fig. 8A vs B; Fig. 8C and D, *I*_S_*/I*_P1_, black vs red bars). Hence, the incapability of ACh to elicit currents after exposure to IVM (Fig. 8B, *I*_P2_ and *I*_P3_; Fig. 8D, *I*_P2_*/I*_P1_ and *I*_P3_*/I*_P1_ in red) strongly indicates that IVM stabilizes, for a long duration, a channel state having an obstructed ion-conduction pathway.

### Does IVM accumulate in the CHO cell plasma membrane during the time of its application?

The capability of a fluorescent ivermectin probe to partition into biological membranes and diffuse laterally in the lipid bilayer was previously demonstrated following its incubation for 30 minutes with muscle vesicle membranes prepared from the parasite *Ascaris sum*^120^. The lipophilic properties of IVM were also demonstrated by incubating IVM with artificial large unilamellar vesicles (LUVs; 6 mM) for 5 minutes, and determining the depletion of IVM from the aqueous solution following a further 20-minutes long ultracentrifugation (50 or 70 percent depletion, depending on the LUVs’ lipid composition)^58^. Hence, one may argue that the irreversible inhibition of the chimeric α7-GluClβR by IVM is due to the accumulation of IVM in the cell membrane and the consequent increase of the effective concentration of the drug around the receptors. To the best of our knowledge, no data is available regarding the partitioning of ivermectin into the plasma membrane of CHO cells in a period as short as the time of IVM application in the current study (no longer than 10 seconds).

To assess the IVM partitioning into the CHO cell plasma membrane during a short time period, we followed the procedure of Silberberg *et al*.^58^ with a few modifications accounting for our experimental conditions. To this end, we incubated 140,000 CHO cells (correspond to 3.2 μM plasma membrane lipids^121^) with 4 μM IVM for either 0, 15 or 300 seconds, pelleted the cells by a brief centrifugation (30 seconds), and analyzed the extent of IVM removal from the aqueous solution (elaborated in the Supplementary Materials and Methods). Supplementary Fig. S3 shows examples of absorption spectra of supernatant obtained after the CHO cells’ sedimentation. These spectra indicate that IVM dissolved in NES containing 0.02% DMSO has an ultraviolet absorption maximum at 245 nm, a wavelength used by others to detect IVM in other solvents^58^,^122^. The maximum absorbance values were used as a measure for the fraction of IVM removal during the incubation times. Figure 7D indicates that following 5 minutes of incubation, ∼24% of IVM was removed from the aqueous solution by the CHO cells. This is less than in artificial LUVs^58^, probably because of the differences in the: membrane lipid composition; IVM hydration conditions; duration of the post-incubation centrifugation; and lipid content (lower in our case as mentioned above). Importantly, within 15 seconds of incubation, no statistically significant amount of IVM was removed from the aqueous solution by the CHO cells (Fig. 7D; e.g. Supplemental Fig. S3). Although these experiments are not sufficiently sensitive to provide the IVM concentration in the CHO cell membrane during the electrophysiological experiments, they indicate that the accumulation of IVM in the membrane is not substantial during the longest IVM applications we employed (10 seconds) (further discussed below).

### Can IVM inhibit homomeric receptors assembled from the *C. elegans* GluClβ subunit?

**Figure 9:**
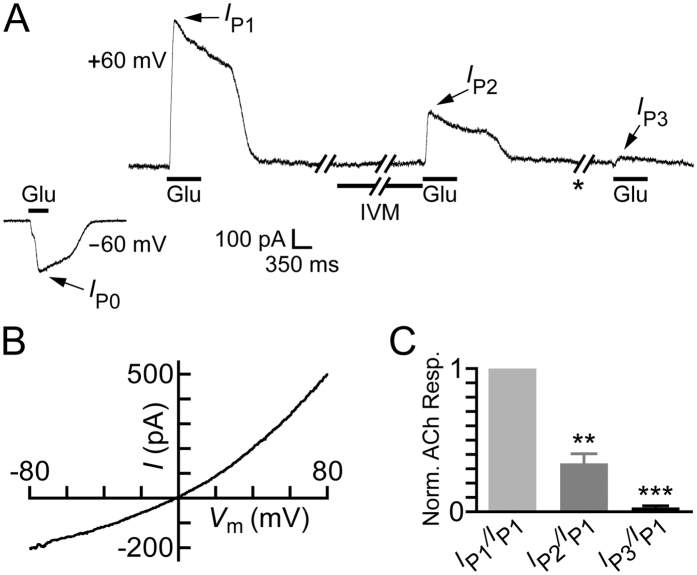
Effect of IVM on homomeric GluClβ receptors. (**A**) Representative current traces obtained by the indicated application protocol. Glu (100 mM) was applied for either 350 ms to obtain peak *I*_P0_ at −60 mV, or 600 ms to obtain peaks *I*_P1_, *I*_P2_ and *I*_P3_ at +60 mV. Note that the responses at both membrane voltages, −60 and +60 mV, start to desensitize appreciably before the end of Glu application. IVM (2 μM) was applied for 10 seconds. Asterisk indicates a 25-sec-long wash between the second and third Glu applications. (**B**) Current-voltage (*I/V*) relations obtained in a different cell than in A upon the application of Glu (100 mM) over a voltage ramp lasting 200 ms. (**C**) Normalized ACh response (y-axis) calculated by the current peak ratios that are indicated below the x-axis. Current ratios were extracted from experiments performed as shown in (**A**). Mean ± SEM of four cells are presented. Paired, two-tailed Student’s *t*-tests indicate that **0.001* < P* < 0.01 and ***P < 0.0002.

The results presented so far show that IVM strongly inhibits the chimeric α7-GluClβR. Since IVM putatively binds in the ion-channel pore domain and this region of the chimeric α7-GluClβR is contributed by the *C. elegans* GluClβ subunit, an inevitable question emerged: would IVM inhibit homomeric receptors assembled from the *C. elegans* GluClβ (GLC-2) subunit ? To answer this question, we attempted to express GluClβR homomers in CHO cells, although in a previous study such functional homomers were rarely expressed in CHO cells and provided small responses to 10 mM Glu^79^. Currently, we have used a more powerful transfection reagent (Supplementary Materials and Methods) and challenged the cells with 100 mM Glu. Four of 46 cells gave reliable currents of hundreds of picoamperes at +60 mV (e.g., Fig. 9A and B). These responses were reminiscent of the behavior displayed by homomeric GluClβRs expressed in *Xenopus* oocytes^4^,^123^. That is, (i) the responses desensitized (e.g., Fig. 9A) as previously shown under a saturating Glu concentration (see Fig. 7A in Etter *et al*.^123^), and (ii) the responses at +60 mV were larger than the responses obtained at −60 mV, with *I*_ + 60mV_/*I*_−60mV_ = 2.8 ± 0.2 (mean ± SEM) (e.g., Fig. 9A, *I*_P1_/*I*_P0_). Accordingly, the *I/V* curve rectifies outwardly (e.g., Fig. 9B), albeit with less steepness than that observed previously in *Xenopus* oocytes^4^. This difference in the *I/V*-curve steepness is partially because we have used approximately equimolar extra- and intracellular Cl^−^ ion concentrations (150.8 mM and 138 mM, respectively), whereas the extracellular and calculated intracellular Cl^–^ ion concentrations used in the experiments with *Xenopus* oocytes were 122.6 mM and ~33 mM, respectively^4^. Notably, the responses of the other 42 cells that we patched were very small (10.8 ± 2.6 pA at +60 mV and 31.1 ± 5.6 pA at −60 mV), providing *I*_ + 60mV_/*I*_−60mV_ = 0.26 ± 0.06; and therefore these responses were considered as nonspecific and irrelevant to the homomeric GluClβR.

Figure 9A and C, also shows that pre-application of 2 μM IVM for 10 seconds leads to the inhibition of ∼67% of the response of the homomeric GluClβRs to 100 mM Glu (*I*_P2_/*I*_P1_). Further application of 100 mM Glu after a wash of 25 seconds provided peak *I*_P3_, which revealed that most of the initial response disappeared (Fig. 9A and C, *I*_P3_/*I*_P1_). Note that, in spite of the efficient perfusion system, which provides a time constant of activation at the millisecond range (33.1 ± 3.7 ms), the Glu-elicited currents slowly returned to baseline upon the application of wash. This slow current decline is probably due to the very high concentration of the applied Glu (100 mM), which is ~260 times the EC_50_ determined in *Xenopus* oocytes (380 μM) for the homomeric GluClβR^4^.

## Discussion

Cys-loop receptor chimeras have long been used to shed light on the interplay between the ligand-binding domain and the ion-channel pore domain^55^,^80^,^81^,^97^,^98^,^124^,^125^,^126^,^127^,^128^,^129^. Here we used a highly expressible, homomeric α7-GluClβ chimeric receptor in an attempt to understand why IVM does not activate the *C. elegans* homomeric GluClβR although it can activate the *C. elegans* heteromeric GluClα/βR. The rationale behind our decision to work with a chimera having the extracellular domain of the α7-nAChR was based on the assumption that the LigBD-pore domain interface might affect IVM action. This assumption emerged because of the following reasons. (i) IVM strongly potentiates ACh-elicited currents in the α7-nAChR^54^. (ii) In the GluClα_cryst_R, the disaccharide and lactone moieties of IVM form van der Waals interactions with the M2-M3 loop^77^ that is located at the LigBD-pore domain interface. (iii) The M2-M3 loop of Cys-loop receptors interacts with the β1β2, Cys and β8β9 loops of the ligBD as well as with the pre-M1 segment to couple agonist binding to channel gating^50^,^83^,^84^,^85^,^86^,^87^,^88^,^89^,^90^,^91^,^92^,^93^,^94^,^95^,^96^,^97^,^98^,^130^,^131^,^132^,^133^,^134^,^135^,^136^. (iv) Incorporation of the GluClβ subunit in GluClR assemblies increases the sensitivity of the receptor to IVM^78^, which suggests that IVM interacts with the GluClβ subunit. We therefore expected that IVM would, at least, potentiate the response of the chimeric α7-GluClβR to ACh. To our great surprise, IVM was found here to strongly inhibit the chimeric α7-GluClβR.

Interestingly, IVM negatively modulates a few other Cys-loop receptors^55^,^137^,^138^. Yet, little is known about the mechanism of the IVM inhibitory phenomenon. Here, based on experiments where IVM was applied prior to ACh, we suggest that IVM can bind to the resting (closed) state of the α7-GluClβR and stabilize this conformation for a long duration. However, we do not exclude the possibility that the stabilized conformation corresponds to a desensitized-like state, since unliganded as well as liganded Cys-loop receptors can interconvert from the resting state to a desensitized state directly without opening; for instance, the neuronal α4β2 nAChR^115^.

IVM binds in the periphery of the ion-channel pore domain of the GluClα_cryst_R^77^, and it was likewise suggested to bind in a homologous peripheral site in the GlyR^137^. Assuming that IVM also binds in the periphery of the ion-channel pore domain of the chimeric α7-GluClβR, then the possibility of competitive inhibition by IVM is excluded. Uncompetitive inhibition, which requires receptor–activator complex formation prior to binding of the inhibitor^139^, is also excluded because IVM can bind already before ACh association with the α7-GluClβR (e.g., Fig. 4). Since non-competitive inhibition can occur with or without the presence of the activator, IVM most likely acts as a non-competitive inhibitor of the chimeric α7-GluClβR (e.g., Figs 4 and 5). Therefore, the IC_50_ determined in Fig. 3 (156 nM) actually corresponds to the inhibition constant (*K*_i_)^139^.

The response of the chimeric α7-GluClβR to ACh declines slowly after reaching to its peak. This decline could be attributed, at least in part, to a loss of electrochemical driving force for Cl^−^ ions, as discussed in the “Results”. However, one cannot exclude the possibility that a fraction of this decline corresponds to channels’ closing in the presence of the agonist due to desensitization. When IVM was added to an already activated receptor population, immediately after the response reached to its peak, the decline of the current became faster and the extent of current loss at the end of the co-application period was larger. As discussed in detail in the “Results” (Fig. 6), the faster and stronger decline in the Cl^−^ conductance and the lesser decrease in [Cl]_i_ and *V*_DF_ during the co-application of ACh with IVM, as compared with the application of ACh alone, strongly suggest that, in the presence of IVM, the receptor adopts a conformation that hinders chloride ion flow. The exponential fits to the specific IVM-dependent Cl^−^-conductance (*G*_IVM_) decay (Fig. 6) allowed us to obtain the affinity of the α7-GluClβR for IVM when the drug is applied after activation (*K*_d_ = 1.4 μM; Fig. 6N). When IVM is applied prior to the activation by ACh, the affinity of the receptor for IVM is nine times higher (*K*_i_ = 156 nM; Fig. 3). Hence, we suggest that IVM can bind to two distinct receptor states. One state is possibly the resting state and the other state is likely to be an open state of the α7-GluClβR. We further suggest that IVM binding to the open state induces a conformational change that results in pore closure. In respect to IVM association with the open state, the aforementioned kinetic model should therefore be revised as follows.


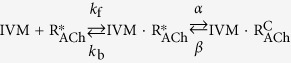


where *k*_f_ and *k*_b_ are the rate constants described above in the first kinetic model, and *α* and *β* are the rates of receptor interconversion between the IVM-bound open state (IVM⋅R*_ACh_) and the IVM-bound non-conducting (closed) state (IVM⋅R^C^_ACh_). We could not determine the receptor interconversion rates, but it is reasonable to assume that if *α* ≫ *β*, then the IVM-bound closed conformation would be stable for a long duration.

Most recently, the three dimensional structure of a homomeric α1 GlyR from the zebrafish (termed GlyR_EM_) was elucidated by electron cryo-microscopy (ECM) in conformations that correspond to the receptor complexed with either glycine alone or with both glycine and IVM^140^. These ECM structures indicate that the ion-channel pore of a receptor occupied by both glycine and IVM is narrower than that of a receptor occupied by glycine alone. Yet, the most constricted portion of the doubly occupied (glycine/IVM) GlyR_EM_ has a diameter of 5_ _Å, which is sufficiently wide to allow Cl^−^ ions to pass^140^. We speculate that the association of IVM with ACh-occupied open α7-GluClβR leads to constriction of the ion-channel pore, but unlike the case of the doubly occupied GlyR_EM_, here the constriction of the α7-GluClβR is not sufficiently wide to allow Cl^−^ ions to flow across the ion-channel pore.

The capability of ACh-elicited currents to recover after the first response to long-term application of ACh alone (Fig. 7A and C, black bars), clearly indicates that no change in the size of the receptor population has occurred. This conclusion excludes the possibility that functional rundown accompanied with permanent inactivation took place. Hence, the gradual recovery seen in Fig. 7A could indicate that reversible desensitization or/and a decrease in *V*_DF_ underlie the lack of current when ACh is reapplied alone 5 to 70 seconds after the end of the initial long-term ACh application (see also Fig. 7C, black bars). We therefore conclude that the small responses to ACh that were obtained after the co-application of ACh with IVM (Fig. 7B and C, red bars) likely correspond to a receptor population that is ‘stuck’ in the above-mentioned channel-constricted conformation.

When IVM is added after the ACh-elicited current reached to its peak, it most likely associates with open receptors. The calculation of the time constant of *G*_IVM_ decay (Fig. 6N) allowed us to determine the residency time of IVM bound to the α7-GluClβR (1/*k*_b_ = ~2.5 sec). This value means that a few seconds after removal of water-soluble IVM from around the cell by a wash, the inhibitory effect should have been weakened due to IVM dissociation and lateral diffusion in the membrane. However, the responses were not even partially recovered after the first exposure of ACh-bound open α7-GluClβRs to IVM (Figs 7 and 8), indicating that there might be an additional binding step that possibly causes trapping of IVM in the pore periphery. Yet, one cannot exclude with absolute certainty the possibility that the irreversibility of the inhibition could be due to a steady, high membranous IVM concentration encountered by the receptors, rather than an irreversible trapping of IVM by the receptors. This possibility arises because, within time, IVM tends to accumulate in lipid bilayers due to its lipophilic properties, and it cannot be removed from the lipidic phase under physiological conditions. Previous studies involving IVM accumulation in artificial lipid bilayers or biological membranes^58^,^120^ are relevant to relatively much longer incubation times than used in the current study (detailed in the “Results”). Indeed, we found that no substantial accumulation of IVM in the membrane of CHO cells is apparent during a 15-sec-long incubation with each other (Fig. 7D and Supplementary Fig. S3). The longest time of exposure to IVM in the electrophysiological experiments performed here was 10 seconds. The perfusion exchange in our system removes soluble small molecules from around the cell within less than a second, as can be judged by the time of the current’s return to the baseline when replacing ACh-containing solution by a wash (407 ± 34 ms, in the experiments represented by Figs 7A and 8A). This means that, after 10 seconds of IVM application, it probably takes less than 1 second to remove all the water-soluble IVM molecules from around the cell, as well. At this time point, no further IVM is available for binding by the receptors, except for those membrane-embedded IVM molecules that, as said above, do not likely reflect a substantial accumulation.

Noteworthy, the effect of IVM might take place due to IVM partitioning into the membrane at the site of its action, or by IVM molecules that partition into the membrane far from their targets and approach their binding sites by lateral movement within the lipid bilayer. Lateral movement of a fluorescently labeled IVM probe in biological membranes was previously determined to be slow, with a lateral diffusion coefficient (*D*_*L*_) of 14⋅10^−10^ cm^2^/s (see page 554 in Martin and Kusel^120^, under “*The lateral mobility of the ivermectin probe*”). In the six experiments represented by Fig. 5A (e.g., red trace), the time between the start of ACh or IVM applications and the onset of their effects was 67 ± 7 or 132 ± 24 milliseconds, respectively. Taking into account the differences in accessibility and complexity between the ACh and IVM binding sites, the two-fold slower onset of effect observed for IVM is reasonable if IVM acts at the site of its partitioning. In this context, it is intriguing to point out that in the GluClα_cryst_ receptor the disaccharide and lactone moieties of IVM interact with the M2-M3 loop^77^ that is putatively situated above the lipid leaflet in all Cys-loop receptors. So, partitioning of IVM into the membrane at the site of action perhaps involves a recognition step that occurs outside of the membrane (still have to be studied).

Collectively, our IVM partitioning experiments and the arguments raised above suggest that the concentration of IVM in the perfused solution approximates the concentration of IVM in the membrane at the end of the 10-sec-long IVM application. Hence, our calculation of *k*_b_ based on the concentration of IVM in the perfused solution is a reasonable approximation. Still, one may argue that since the washes do not extract IVM from the membrane, the irreversibility may reflect an exchange between receptor-bound and membrane-resident IVM molecules. The counter argument is based on Fig. 6K, which indicates that 740 milliseconds after the application of 0.5 μM IVM, there is already a statistically significant IVM-dependent decrease in the chloride conductance by 20 ± 2% (*P* = 0.0005, paired, two-tailed Student’s *t*-test). This IVM concentration (0.5 μM) does not exert the maximal inhibitory effect according to the declining rate of the chloride conductance (Fig. 6). Yet, we did not observe even a partial recovery of the initial response following IVM removal from the external solution and the subsequent ACh applications (Figs 7B and 8B); which likely indicates that IVM is trapped in its binding site(s) for a long duration.

In conclusion, the results presented here suggest that IVM can bind to both, the resting and active states of the α7-GluClβR. After initial binding of the drug to the active (open) state, it accelerates pore constriction to a degree that impedes Cl^−^ ion flow through the ion-channel pore. We suggest that at the time the pore becomes constricted in a non-conductive conformation, IVM is likely trapped between the transmembrane segments for a long duration and it reciprocally locks the pore-lining segments. As such, gating motions necessary for re-opening of the ion-channel pore are prevented.

So far, it was not known why homomeric receptors assembled from the *C. elegans* GluClβ (GLC-2) subunit could not be activated by IVM^4^,^7^,^8^,^76^. The current study provides an answer to this question by demonstrating that IVM actually inhibits the responses of the *C. elegans* homomeric GluClβR to Glu (Fig. 9). IVM likely binds in the periphery of the ion-channel pore domain, between adjacent subunits of the homomeric GluClβR, akin to the case of the GluClα_cryst_R. If so, IVM is accommodated by the homomeric GluClβR at β/β intersubunit interfaces. In a previous study, we inferred that the subunits of the *C. elegans* heteromeric GluClα(GLC-1)/β(GLC-2) receptor are arranged in an anticlockwise β-α-β-α-α fashion, as viewed from the extracellular milieu^79^. This subunit arrangement does not have β/β intersubunit interfaces. Hence, we further hypothesize that in the IVM-activatable heteromeric GluClα/βR, the subunit arrangement precludes potential restraints that could have emerged by IVM binding at such β/β interfaces.

## Materials and Methods

“Homology modelling”, “DNA construct and preparation of cells for electrophysiological experiments” and “Whole-cell patch clamp recordings” are described in the Supplementary Materials and Methods.

### Data analysis

Analyses were performed using the Clampfit 10 program implemented in pClamp 10, and the GraphPad Prism software.

Dose-response curves were fitted to the data points by a nonlinear regression using the Hill equation 1,


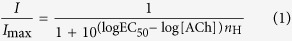


where *I* is the current response, *I*_max_ is the maximal current response, EC_50_ is the agonist effective concentration that elicits 50% of maximal current response, [ACh] is the concentration of acetylcholine, and *n*_H_ is the Hill coefficient.

In the case of inhibition of the current response, curves were fitted to the data points by a nonlinear regression using the Hill equation 2,


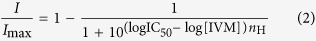


where *I* is the current response, *I*_max_ is the maximal current response, IC_50_ is the IVM concentration that inhibits 50% of the current response, [IVM] is the concentration of ivermectin, and *n*_H_ is the Hill coefficient.

The declining phase of the currents was fitted with an exponential time course to give the time constant of current decline by using equation 3,


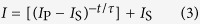


or its bi-exponential variant where appropriate, using data that relates to ∼90% of the period between the peak of the current and the termination of a typical 5.5- or 9.6-sec-long ACh (or ACh + IVM) application (i.e., the fitting was performed from the point of IVM addition). *I* is the current, *I*_P_ is the peak of the current, *I*_S_ is the steady state current, *t* is the time, and *τ* is the time constant of current decline.

To calculate the changes in the intracellular concentration of Cl^−^ ions ([Cl]_i_) in the voltage-ramp experiments, the Nernst equation (4) was used as follows:


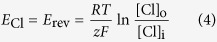


where *E*_Cl_ is the Nernst (equilibrium) potential for Cl^−^ ions; *E*_rev_ is the measured reversal potential; *R* and *F* are the gas and Faraday constants, respectively; *T* is the absolute temperature (298.15 K); *z* is the valance of a Cl^−^ ion (−1); [Cl]_o_ is the extracellular concentration of Cl^−^ ions; and [Cl]_i_ is the intracellular concentration of Cl^−^ ions.

Chord conductance (*G*) was calculated by using equation 5 as follows:


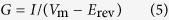


where *G* is the chloride conductance, *I* corresponds to the current measured at the respective membrane voltage (*V*_m_), and *E*_rev_ is the reversal potential.

The relationship between the conductance values and time was plotted for each cell, and the time constant of conductance decay was determined by fitting an exponential curve to the data points using equation 6 as follows:


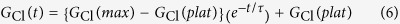


or its bi-exponential variant where appropriate. *G*_Cl_(*t*) is the chloride conductance at a given time; *G*_Cl_(*max*) and *G*_Cl_(*plat*) are the chloride conductance values at the maximum and the plateau, respectively; *t* is the time; and *τ* is the time constant of the chloride-conductance decay to the plateau.

To calculate the IVM association (*k*_f_) and dissociation (*k*_b_) rate constants, a curve was fitted to the 1/*τ*_IVM_ values by a linear regression using equation 7 as follows:


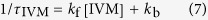


where *τ*_IVM_ is the time constant of the IVM-dependent Cl^−^-conductance decay, and [IVM] is the concentration of IVM.

## Additional Information

**How to cite this article**: Degani-Katzav, N. *et al*. Trapping of ivermectin by a pentameric ligand-gated ion channel upon open-to-closed isomerization. *Sci. Rep.* **7**, 42481; doi: 10.1038/srep42481 (2017).

**Publisher's note:** Springer Nature remains neutral with regard to jurisdictional claims in published maps and institutional affiliations.

## Supplementary Material

Supplementary Information
