# Supplementary material for: Trapping of ivermectin by a pentameric ligand-gated ion channel upon open-to-closed isomerization
Source: Sci Rep. 2017 Feb 20;7:42481. doi: 10.1038/srep42481 (PMC5317004; doi:10.1038/srep42481)
Supplement: Supplementary Information [file srep42481-s1.pdf]

Supplementary Information

Trapping of ivermectin by a pentameric ligand-gated ion channel upon open-to-closed isomerization

N. Degani-Katzav, M. Klein, M. Har-Even, R. Gortler, R. Tobi and Y. Paas*

Supplementary Materials and Methods

Homology modelling

The structural model of the chimeric α7-GluClβR was built based on the methodologies published previously[1,2], and the sequence of the chimeric subunit is presented in Supplementary Figure S1.

DNA construct and preparation of cells for electrophysiological experiments

The cDNA encoding the α7-GluClβ chimeric subunit was generated as described previously[3], and was cloned into a pcDNA3.1 vector.

Chinese hamster ovary cells (CHO-K1, ATCC via Biological Industries Israel, Beit Haemek Ltd.) were cultured as performed previously[2] in Dulbecco's Modified Eagle Medium (DMEM) containing 10% fetal calf serum (Biological Industries Israel Beit Haemek Ltd.), 2 mM glutamax, 100 Units/ml penicillin G and 100 µg/ml streptomycin, and grown in 5% CO2 at 37°C under 90-95% humidity. CHO cells were seeded on glass coverslips (13-mm diameter) placed in a 24-multiwell plate. For the expression of the α7-GluClβR, cells were transiently transfected with the pcDNA3.1 plasmid containing the receptor's ORF (200-600 ng per well) using transit-LT1 transfection reagent (Mirus, USA), according to the manufacturer's protocol. pIRES-CD8 plasmid (200 ng per well) was also added to enable visualization of the expressing cells by beads coated with anti-CD8 antibodies (Invitrogen). For the expression of GluClβ homomers, we used the pcDNA3.1 plasmid containing the GluClβ-subunit ORF (600-1000 ng per well), pIRES-CD8 plasmid (200 ng per well) and the X-tremeGENE HP DNA Transfection Reagent (Roche Life Science). Recordings were performed 48-72 hours following transfection.

Whole-cell patch clamp recordings

Recordings were performed in the aforementioned CHO cells, as previously described[1,2]. Whole-cell patch clamp experiments were performed with normal external solution (NES) contained (in millimolar): 140 NaCl, 2.8 KCl, 2 $CaCl_2$, 2 $MgCl_2$, 10 glucose and 10 HEPES, adjusted to pH 7.35 with NaOH (310 mOsM). The pipette solution contained

(in millimolar): 130 KCl, 4 $MgCl_2$, 4 Na_2ATP, 1 EGTA and 10 HEPES, adjusted to pH 7.35 with KOH (290 mOsM). The osmolarity of these solutions was maintained by adding sucrose. The electrode resistance was 6-10 MΩ when filled with the pipette solution. External solutions were applied onto the cell by using the VC-77SP fast-step system (Warner Instruments, CT, USA) combined with N_2 pressure of 3-4 psi to produce laminar flow of the external solution onto the patched cell. All measurements were performed at 25 °C. The currents were measured with an Axopatch 200B patch-clamp amplifier (Molecular Devices, Sunnyvale, CA) and a Digidata 1440A interface (Molecular Devices). Acquisition of recording data was performed at 2.5 kHz and recordings were low-pass filtered at 1 kHz, through a four-pole Bessel filter. The pClamp 10 software (Molecular Devices) was used for data acquisition.

To determine the chloride conductance and the reversal potential during current decline, the following procedure was employed. First, short ACh applications (30 μM for 350 msec) were repeated at a holding potential of -60 mV until consecutive equal current amplitudes were obtained. Then, currents were elicited by a long-term (9.6 sec) application of ACh (30 μM), and after the current reached to its peak, 13 voltage ramps (r1 to r13) were applied during the declining phase of the current. A ramp included a step from -60 mV to -80 mV for 10 ms, followed by 200-ms-long voltage ramp from -80 mV to +20 mV and a step back to -60 mV. At the end of this 9.6-sec-long ACh application, a minute of wash was applied. Then, the long-ACh-application-experiment was re-conducted, but after the current reached to its peak, IVM (at either 0.5, 2 or 4 μM) was co-applied with ACh (30 μM). Finally, following a long wash, the same experiment was conducted without IVM, to assess the extent of recovery from the IVM effect. Routinely, leak currents were obtained by the same protocol, before exposure of the cell to ligands. The leak currents were subtracted from currents obtained in the presence ACh or ACh + IVM. Because of technical limitations, each experiment that combined voltage ramps with long ACh (or ACh + IVM) application had to be conducted by separate protocols that are linked to follow one another. The two successive protocols had a time lapse of about 1 sec between protocols. Since the protocols' exchange was executed between the third (r3) and the forth (r4) voltage ramps, the time between these two ramps is longer than the time between the other ramps. Note that ACh application, without or with IVM, is continuous since the appropriate perfusion valve was opened manually during the time lapse. Reversal potential (E_{rev}) and chord conductance at -65 mV and +15 mV were extracted from the output currents of the voltage ramps.

The voltage ramps in the experiments with the homomeric GluClβR were performed as follows. Glu (100 mM) was applied for 420 ms. 200 ms after the application started (which is

also after the current reached to its peak at -60 mV), the voltage was stepped from -60 mV to -80 mV for 20 ms followed by a 200-ms-long voltage ramp ranging from -80 mV to +20 mV.

Assessment of time-dependent IVM partitioning into the CHO cell membrane

CHO cells were cultured as above in 100-mm cell-culture dishes (Corning). The adherent cells were washed twice with 10 ml phosphate buffered saline (PBS) per dish, and then harvested with 2 ml of Recombinant Trypsin EDTA Solution (Biological Industries Israel, Beit Haemek Ltd.). The trypsin solution containing the harvested cells was diluted by fivefold in PBS and the cells were subjected to centrifugation at 400 g for 2.5 minutes. The pelleted cells were suspended in 10 ml ice-cold NES, and pelleted again, as above. This step was repeated once more and the cells were finally re-suspended in ice-cold NES to give 280,000 cells per ml.

A solution of NES containing 8 μM IVM and 0.04% DMSO was freshly prepared in a 50 ml polypropylene tube that was preincubated with 8 μM IVM for 2 hours, to minimized the loss of IVM from the solution during the experiments due to non-specific adhesion of IVM to the surface of the tube. Then, the association of IVM with the cells was measured in 2-ml Eppendorf tubes at room temperature as follows. Half ml of the aforementioned IVM solution was added to half ml of NES containing 140,000 CHO cells, mixed rapidly by a gentle vortex and subjected to 3,000 g centrifugation for 30 seconds, either immediately after the mixing (i.e., 0 second) or after incubations for 15 or 300 seconds. Note that: (i) each time point was accompanied with a reference containing the same cell mixture (including DMSO) but devoid of IVM, and (ii) each time point, which included only two tubes (–/+ IVM), was examined separately; so, the lag between the mixing and the start of incubation was 5 seconds. Following the sedimentation of the cells, the pellet was clearly seen by the eye allowing us to readily remove 0.75 ml of the supernatant for analysis in a Ultrospec 2100 Pro UV/Visible Spectrophotometer (GE Healthcare). This analysis included measurements of absorption spectra from 190 to 400 nm wavelengths (e.g., Supplementary Fig. S3). The maximum absorbance of IVM is at 245 nm wavelength. Note that the zero-time point and the aforementioned reference (– IVM) exclude uncertainties that could have emerged due to partitioning of IVM and DMSO into the lipid bilayer during the 30-sec-long centrifugation, and the potential loss of IVM from the solution because of non-specific adhesion to the surface of the 2-ml Eppendorf tubes.

Supplementary References

1 Bar-Lev, D. D., Degani-Katzav, N., Perelman, A. & Paas, Y. Molecular dissection of Cl--selective Cys-loop receptor points to components that are dispensable or essential for channel activity. *J Biol Chem* **286**, 43830-43841 (2011).

2 Degani-Katzav, N., Gortler, R., Gorodetzki, L. & Paas, Y. Subunit stoichiometry and arrangement in a heteromeric glutamate-gated chloride channel. *Proc Natl Acad Sci U S A* **113**, E644-653 (2016).

3 Sunesen, M. *et al.* Mechanism of Cl- selection by a glutamate-gated chloride (GluCl) receptor revealed through mutations in the selectivity filter. *J Biol Chem* **281**, 14875-14881 (2006).

Supplementary Figures

```
MGLRALMLWLLAATGLVRESLQGEFQRKLYKELLKNYNPLERPVANDSQPLTVYFTLSLM
QIMDVDEKNQVLTTNIWLQMYWTDHYLQWNVSEYPGVKNVRFPDGLIWKPDILLYNSADE
RFDATFHTNVLVNSSGHCQYLPPGIFKSSCYIDVRWFPFDVQKCNLKFGSWTYGGWSLDL
QMQEADISGYISNGEWDLVGIPGKRTESFYECCKEPYPDITFTVIIRRRFSYYLVQLYAP
TTMIVIVSWVSFWIDLHSTAGRVALGVTTLLTMTTMQSAINAKLPPVSYVKVVDVWLGAC
QTFVFGALLEYAFVSYQDSVRQNDRSREKAARKAQRRREKLEMVDAEVYQPPCTCHTFEA
RETFRDKVRRYFTKPDYLPAKIDFYARFVVPLAFLAFNVIYWVSCLIMSANASTPESLV
```

Supplementary Figure S1. Amino acid sequence of the chimeric α7-GluClβ subunit. The signal peptide is highlighted in grey. The extracellular amino-terminal segment, which was taken from the chick nAChR α7 subunit (UniProtKB accession no. P22770), is highlighted in reddish color. The two consecutive isoleucine amino acids shown in blue, replace the threonine and methionine of the α7-nAChR at these two positions, as they appeared to be important for functional coupling of the neurotransmitter-binding domain of α7 and the ion-channel domain of another successful chimera, the α7-V201-5HT3AR. The sequence that was taken from the *C. elegans* GluClβ subunit (UniProtKB Q17328) is highlighted in green. Colors match the colors used in Fig. 1A. The pore-lining segment, M2, is underlined.

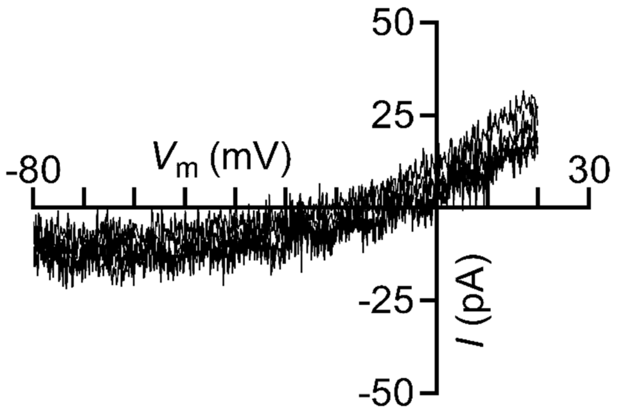

Supplementary Figure S2. Current-voltage (*I/V*) relations extracted from voltage ramps r1, r2, r3, r4 and r13 (as performed in Fig. 6C). The I/V relations represent the same cell shown in Fig. 6 A and B. This cell was exposed to 30 µM ACh alone after it experienced the co-application of ACh plus IVM (in Fig. 6B). Note that the current scale is in picoampere.

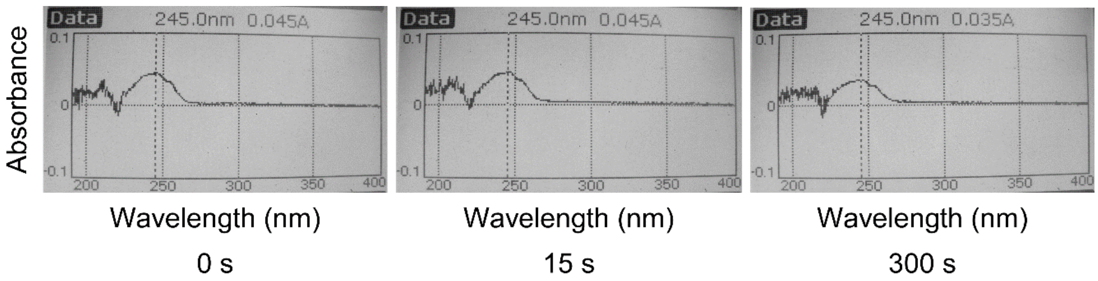

Supplementary Figure S3. Representative absorbance spectra of supernatant samples containing ivermectin (IVM) that was not incorporated in the membrane of Chinese hamster ovary (CHO) cells during the incubation with each other. Times of IVM incubation with the cells are indicated below the panels. The dotted vertical lines indicate the absorbance at 245 nm, and the absorbance values (A) are provided on top of the images. Images correspond to pictures of the spectrophotometer's screen taken with a manual Canon PowerShot S100 camera. Details regarding the reference wave scan are provided in the Supplementary Materials and Methods under "Assessment of time-dependent IVM partitioning into the CHO cell membrane".
